# Workarounds in Electronic Health Record Systems and the Revised Sociotechnical Electronic Health Record Workaround Analysis Framework: Scoping Review

**DOI:** 10.2196/33046

**Published:** 2022-03-15

**Authors:** Vincent Blijleven, Florian Hoxha, Monique Jaspers

**Affiliations:** 1 Center for Marketing & Supply Chain Management Nyenrode Business Universiteit Breukelen Netherlands; 2 Center for Human Factors Engineering of Health Information Technology Amsterdam Public Health Research Institute Amsterdam UMC Amsterdam Netherlands

**Keywords:** electronic health records, electronic medical records, framework, patient safety, unintended consequences, usability, workarounds, workflow

## Abstract

**Background:**

Electronic health record (EHR) system users devise workarounds to cope with mismatches between workflows designed in the EHR and preferred workflows in practice. Although workarounds appear beneficial at first sight, they frequently jeopardize patient safety, the quality of care, and the efficiency of care.

**Objective:**

This review aims to aid in identifying, analyzing, and resolving EHR workarounds; the Sociotechnical EHR Workaround Analysis (SEWA) framework was published in 2019. Although the framework was based on a large case study, the framework still required theoretical validation, refinement, and enrichment.

**Methods:**

A scoping literature review was performed on studies related to EHR workarounds published between 2010 and 2021 in the MEDLINE, Embase, CINAHL, Cochrane, or IEEE databases. A total of 737 studies were retrieved, of which 62 (8.4%) were included in the final analysis. Using an analytic framework, the included studies were investigated to uncover the rationales that EHR users have for workarounds, attributes characterizing workarounds, possible scopes, and types of perceived impacts of workarounds.

**Results:**

The SEWA framework was theoretically validated and extended based on the scoping review. Extensive support for the pre-existing rationales, attributes, possible scopes, and types of impact was found in the included studies. Moreover, 7 new rationales, 4 new attributes, and 3 new types of impact were incorporated. Similarly, the descriptions of multiple pre-existing rationales for workarounds were refined to describe each rationale more accurately.

**Conclusions:**

SEWA is now grounded in the existing body of peer-reviewed empirical evidence on EHR workarounds and, as such, provides a theoretically validated and more complete synthesis of EHR workaround rationales, attributes, possible scopes, and types of impact. The revised SEWA framework can aid researchers and practitioners in a wider range of health care settings to identify, analyze, and resolve workarounds. This will improve user-centered EHR design and redesign, ultimately leading to improved patient safety, quality of care, and efficiency of care.

## Introduction

Electronic health record (EHR) systems are the backbone of modern health care organizations. This is in pursuit of promising gains in patient safety, quality of care, efficiency, and control of spiraling costs by enabling value-based reimbursements. However, realizing these expected benefits is far from a given value. Over the years, an overwhelming number of studies have reported that EHRs have led to a multitude of unintended consequences. Examples include potential patient harm resulting from bad EHR usability [1,2]; increased odds of burnout of health care professionals [3,4]; physicians experiencing stress [5]; users spending an equal amount of time on *desktop medicine* as they spend on having face-to-face interaction with patients [6,7]; extensive *copy and paste* practices of patient notes leading to note bloating, internal inconsistencies, and errors [8]; and the unavailability of complete clinical information at the point of care [9].

Many causes of unintended consequences of EHR use can be traced back to discrepancies between the behavior, intentions, and expectations of EHR users and the workflows dictated by EHRs [10-15]. When EHR users experience workflow mismatches, they often create workarounds [16]. Workarounds are practices that handle exceptions to normal workflow [17] and do not follow the rules, assumptions, workflow regulations, or intentions of systems designers [18]. Although workarounds allow EHR users to proceed in accomplishing tasks in their preferred way (with or without the EHR), research shows that workarounds frequently jeopardize the safety, quality, and efficiency of care [19]. Given their common adverse effects, workarounds are valuable points of departure for improving the EHR design and redesign.

Blijleven et al [20] developed the Sociotechnical EHR Workaround Analysis (SEWA) framework for identifying, analyzing, and subsequently resolving EHR workarounds. The framework was inspired by the Systems Engineering Initiative for Patient Safety (SEIPS) framework [21]. The SEWA framework incorporates four angles: the different rationales EHR users have for creating workarounds (eg, memory aid and required data entry option missing), the stakeholders affected by a workaround (eg, patient and health care professional), the impact of a workaround (eg, on safety and efficiency), and inherent attributes of workarounds (eg, unavoidable, repetitive, and cascading).

The SEWA framework [20] was based on approximately 200 hours of audiovisual material of user–EHR interaction and semistructured follow-up interviews in a single large case study in an academic hospital setting [19,22]. However, the authors argued that the applicability of the framework in other contexts might be limited, such as in nonacademic hospitals or in hospitals where paper-based workarounds (eg, for ordering drugs) are still allowed. Therefore, they recommended validation, refinement, and enrichment of the framework by incorporating workarounds and related rationales, attributes, possible scopes, and types of consequences identified in other EHR workaround–related research and clinical contexts.

To address these shortcomings, a scoping literature review was performed to identify and map the available evidence on EHR workarounds [23]. This paper presents a revised version of the SEWA framework, with rationales, attributes, possible scopes, and types of impact described in workaround-related studies in the EHR, electronic medical record, and computerized physician order entry domains in primary, secondary, and tertiary care contexts published between 2010 and 2021.

## Methods

### Search Strategy

The MEDLINE, Embase, CINAHL, Cochrane, and IEEE databases were searched for relevant studies. We included original, full papers of research with empirical data and conference papers if there were no full papers published in the same study. *Gray literature*, such as books, was not considered. The search queries included the keywords *EHR*, *electronic health record*, and *workaround(s)* and their synonyms. As the aim was to identify new rationales, attributes, consequences, and scopes of EHR workarounds for the enrichment of the SEWA framework, we defined the searches as broad as possible. Pilot literature searches were conducted to check the appropriateness of the queries. During the pilot searches, the term *workflow* was used as a possible synonym for workarounds. The inclusion of this term led to a much larger pool of possible studies. However, most of these studies were focused on care processes that have no relation with EHR use and were thus, out of scope. Therefore, this term was excluded from search queries. Furthermore, to include the complete spectrum of possible EHRs, a combination of the terms *health/medical/patient/health care/clinical record* and *electronic/digital/online* was used. The results of this pilot evaluation were used to adjust the queries. The used queries are shown in Table 1.

**Table 1:** Search queries used for the scoping review.

Date of search	Database	Query
April 9, 2021	MEDLINE	([([([(((((health record*) OR medical record*) OR patient record*) OR health care record*) OR clinical record*) AND electronic] OR digital) OR digitized] OR online) OR online] OR [([Electronic Health Records (MeSH Terms)] OR electronic health record*) OR EHR] OR [([Medical Records Systems, Computerized (MeSH Terms)] OR computerized patient record) OR computerised patient record]) AND ([(workaround*) OR work around*] OR workaround*)
April 9, 2021	Embase	(workaround OR workaround* OR workaround OR workaround*) AND ([(health record* OR medical record* OR patient record* OR health care record* OR clinical record*) AND (electronic OR digital OR online OR online OR digitized OR digitised)] OR [electronic health record* OR ehr OR electronic medical record* OR emr] OR [computerized patient record OR computerised patient record])
April 9, 2021	CINAHL	(workaround OR work around OR workarounds) AND ([(health record OR medical record OR patient record OR health care record OR clinical record) AND (electronic OR digital OR [online OR online] OR [digitized OR digitised])] OR [electronic health record* OR EHR OR electronic medical record* OR EMR] OR [computerized patient record OR computerised patient record])
April 9, 2021	IEEE	([([([(workaround*) OR work around*] OR workaround*)])] AND [([health record OR medical record OR patient record OR health care record OR clinical record] AND [electronic OR digital OR (online OR online) OR (digitized OR digitised)]) OR (electronic health record* OR EHR OR electronic medical record* OR EMR) OR (computerized patient record OR computerised patient record)])
April 9, 2021	Cochrane	(workaround*): ti, ab,kw OR (work-around*): ti, ab, kw OR (work around*): ti, ab, kw AND ([(electronic health record*): ti, ab, kw OR (health record*): ti, ab, kw OR (medical record*): ti, ab, kw OR (patient record*): ti, ab, kw OR (health care record): ti, ab, kw OR (EHR): ti, ab, kw OR (EMR):ti, ab, kw OR (clinical record):ti, ab, kw OR ([computerized patient record]: ti, ab, kw OR [computerized patient record]: ti, ab, kw)] AND [electronic]: ti, ab, kw OR [digital]: ti, ab, kw OR [online]: ti, ab, kw OR [online]: ti, ab, kw OR [digitized]: ti, ab, kw OR [digitised]: ti, ab, kw)

### Selection Criteria

The inclusion and exclusion criteria were chosen through discussions among the reviewers (FH, VB, and MJ). As the focus of this scoping review was on workarounds in EHR use, it was decided to exclude studies focused on barcode medication administration systems as these systems serve only 1 purpose and cover only a small part of the medication process. Furthermore, the choice was made to exclude research focused on EHR functionalities other than those aimed at supporting the clinical process. To ensure data quality, a study was excluded if the research methods were not reported or in case the study had not been peer reviewed. Furthermore, research published before 2010 was excluded as EHRs have undergone significant changes and improvements over the years. Finally, the inclusion and exclusion criteria were chosen.

The study inclusion criteria were as follows:

1. The health care setting of the study must be either ≥1 of primary, secondary, or tertiary care.
2. Workarounds were studied or reported in the context of EHR use.
3. The article was published between 2010 and 2021.

Studies were excluded if they met any of the following criteria:

1. The research focused on EHR functionalities other than those aimed at supporting within the clinical process.
2. The research focused on a barcode administration functionality.
3. The article was not written in English.
4. There was no access to the full-text article.
5. The article was not peer reviewed.
6. The research methods were not reported.

### Article Selection

**Figure 1:**
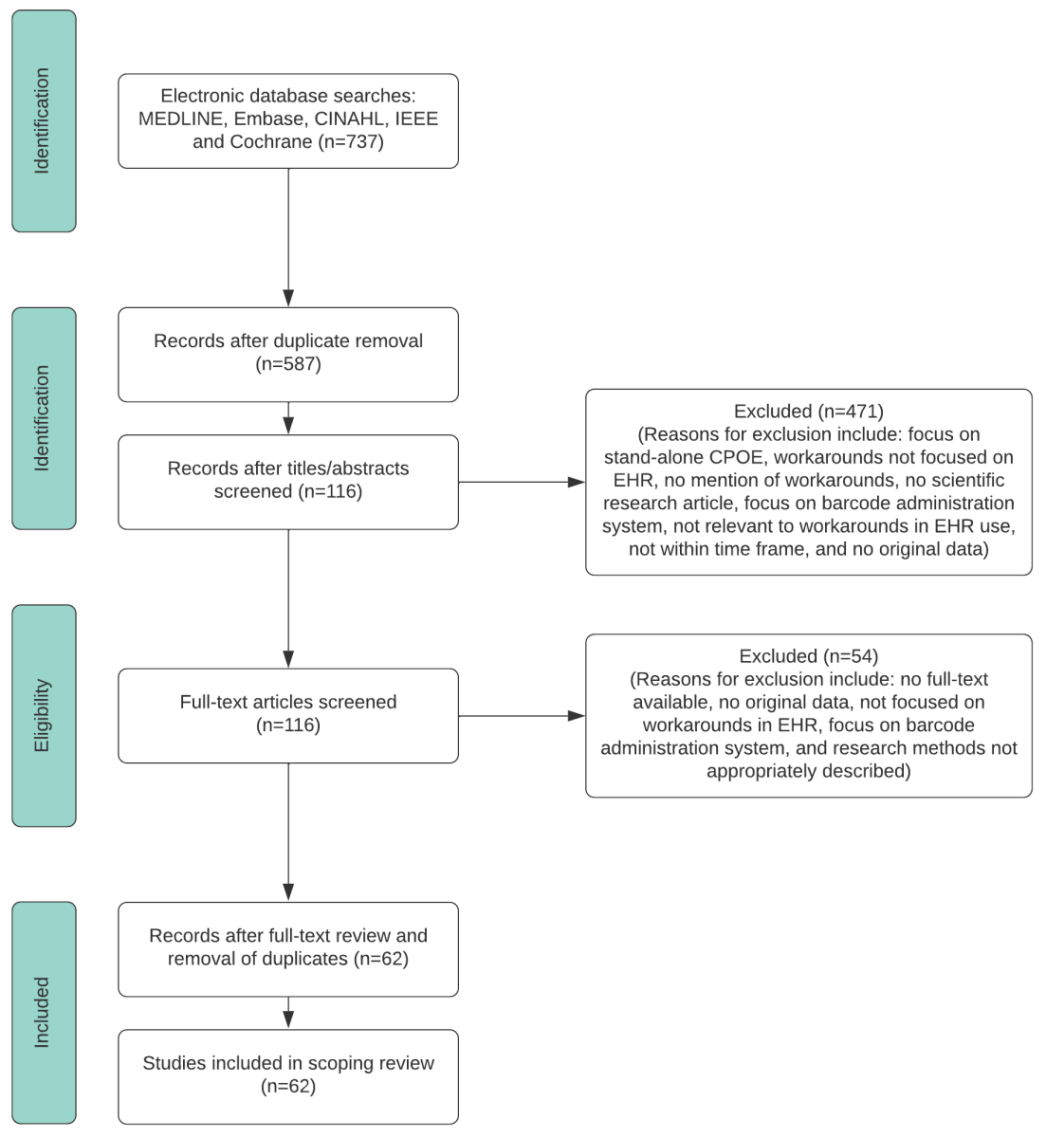
PRISMA (Preferred Reporting Item for Systematic Reviews and Meta-Analyses) flowchart of the study selection process. CPOE: computerized physician order entry; EHR: electronic health record.

A literature search was conducted in April 2021. A total of 737 potentially relevant studies were retrieved from our initial search of electronic databases, more specifically MEDLINE (263/737, 35.7%), Embase (121/737, 16.4%), CINAHL (89/737, 12.1%), IEEE (58/737, 7.9%), and Cochrane (206/737, 27.9%). The results of the study selection process are shown in the PRISMA (Preferred Reporting Item for Systematic Reviews and Meta-Analyses) flowchart in Figure 1.

The retrieved 737 studies were uploaded to EndNoteX9 (Clarivate), in which duplicates were first removed by both using EndNoteX9 and by performing a manual check (Figure 1). This led to 79.6% (587/737) of unique studies. These studies were reviewed by two independent reviewers (FH and VB). The 2 reviewers first independently screened the titles and abstracts of the eligible papers to evaluate whether they met the inclusion criteria. Of the 587 studies, 116 (19.8%) studies met the inclusion criteria, and 471 (60.2%) studies were excluded (because of, for example, workarounds not being focused on the EHR, not being a scientific research article, and no workarounds mentioned). Afterward, the reviewers independently screened the full texts of these 116 studies, leading to 62 (53.4%) included studies and 54 (46.6%) excluded studies (eg, no full-text available and methods inappropriately described). After each screening phase, the two reviewers (FH and VB) discussed their findings. The next screening phase was conducted only if a consensus was reached between the 2 independent reviewers. If a disagreement between the 2 reviewers could not be resolved by discussion, a third independent reviewer (MJ) was involved. After consensus was reached, interrater reliability was reported by calculating the Cohen κ. The interrater agreement was also calculated to show the extent to which the reviewers were able to reconcile through discussion [24]. For the first round (title and abstract screening), the Cohen κ value was 0.958, and the interrater agreement value was 0.985. For the second round (full-text screening), the Cohen κ value was 0.930, and the interrater agreement value was 0.966.

### Data Analysis of Included Articles

Descriptive data from the included articles, such as title, authors, year of publication, study setting, functionalities of EHR studied, and research methods used, were captured in a generic overview per study (Multimedia Appendix 1). Workaround-related data from the included articles, such as workaround rationales, attributes, consequences, and scope, were captured in an analytic frame per study (Multimedia Appendix 2).

The data extracted from the included articles were compared with the SEWA framework on a study-by-study basis. In doing so, SEWA was supplemented with new rationales, attributes, possible scopes, and types of impact of EHR workarounds that were not previously included. After the analysis was completed, an updated (graphical) version of the SEWA framework was created.

## Results

### General Characteristics

The general characteristics of the 62 studies are shown in Table 2. There was an approximately even split in studies published between 2010 and 2015 and between 2016 and 2021. The study settings were almost equally distributed, with most (23/62, 37%) being set in tertiary care, such as academic hospitals and special care units. The largest group of studies (28/62, 45%) focused their research on the EHR overall. Of the 62 studies, 17 (27%) studied medication-related functionalities or EHR-integrated systems, such as computerized physician order entries. Approximately half (28/62, 45%) used or included a combination of physicians, nurses, and other staff such as pharmacists and administrative personnel as participants. Of the 62 studies, 26 (42%) used a combination of methods such as observations, interviews, and questionnaires, 15 (24%) used interviews as the sole method, 5 (8%) solely used questionnaires, 7 (11%) solely used observational methods, and 9 (15%) used other methods such as think-aloud protocols and documentation analysis.

**Table 2:** General characteristics of the included studies (N=62).

Study characteristics		Values, n (%)
**Year of publication**		
	2010-2015	30 (48)
	2016-2021	32 (52)
**Study setting**		
	Primary care	18 (29)
	Secondary care	21 (34)
	Tertiary care	23 (37)
**Functionalities of EHR^a^ studied**		
	Medication-related (eg, prescribing and CPOE^b^)	17 (27)
	Documentation	8 (13)
	Overall EHR	28 (45)
	Others (eg, alert systems and authentication process)	9 (15)
**Type of population**		
	Physicians	9 (15)
	Nurses	13 (21)
	Others (eg, pharmacists or administrative staff such as managers, assistants, secretary, or not mentioned)	12 (19)
	Combination of users	28 (45)
**Methods**		
	Observations	7 (11)
	Interviews	15 (24)
	Questionnaires	5 (8)
	Others (eg, think-aloud and documentation analysis)	9 (15)
	Combination of ≥1 observation, interview, questionnaire, or other	26 (42)

^a^EHR: electronic health record.

^b^CPOE: computerized physician order entry.

### Validation, Refinement, and Enrichment of the SEWA Framework

#### Overview

Evidence for the work system components, rationales, attributes, type of impact, and possible scopes contained in the original SEWA framework was found in the included studies. Moreover, we refined and enriched the original framework with 7 rationales, 4 attributes, and 3 types of impact. The following subsections elaborate on the work system components, rationales, attributes, possible scopes, and types of impact.

#### Work System Components

Support for all 5 work system components was found in the included studies, as shown in Table 3. No new work system components were identified. However, we made 1 change to the work system component *EHR system*, which we renamed to *EHR system and related technology*. The latter was incorporated to also cover workarounds stemming from the use of technology other than the EHR but used in parallel with the EHR, such as scanners [25].

**Table 3:** Overview of work system components and related included studies.

Work components	Description	Studies
Person(s)	Health care professionals developing and using EHR^a^ workarounds	[20,26-28]
EHR system and related technology	The EHR and related information technology used by health care professionals	[20,25-27,29-31]
Organization	Organizational conditions (eg, care directives and hospital policies) under which clinical tasks and EHR use are performed	[20,27,28,30,31]
Physical environment	The environment (eg, outpatient examination room and inpatient ward) and its conditions (eg, lighting and noise) in which clinical tasks are conducted by health care professionals	[20,26,27]
Task(s)	Clinical tasks performed by health care professionals	[20,26,28,30-32]

^a^EHR: electronic health record.

#### Rationales

The rationales for workarounds contained in the original SEWA framework were confirmed in many studies. In addition, 7 new rationales were identified.

Under the work system component *person(s)*, one rationale was added: *trust* (Table 4). Multiple studies reported that users created workarounds because of insufficient trust in the (new) system or its capabilities while frequently maintaining trust in older systems (replaced by the EHR). The related causes of a lack of trust are a lack of perceived usefulness of the (new) system and insufficient confidence in (completeness) of the data available in the EHR [33-39]. The description of the rationale *awareness* has been refined to also cover awareness of the information needs of patients and not just of colleagues [40]. Likewise, the description of the rationale *social norms* has been refined to make cultural [30,41] and collaborative [27,42] aspects more explicit.

Although extensive support in the included studies was found for all rationales under the work system component *EHR system and related technology*, except *patient data specificity,* four additional rationales were identified: *data integration*, *enforced actions*, *data quality*, and *interoperability* (Table 5). The description of the pre-existing rationale *technical issues* has been refined to cover technical issues related to ancillary technology used in conjunction with the EHR.

Multiple studies provide support for all rationales under the work system component *organization* except for the rationale *data migration policy* (Table 6). No new rationales were identified.

Although support was found for the pre-existing rationales under *task(s)*, one rationale was added: *task complexity* (Table 7). Approximately 3% (2/62) of studies described that the EHR does not always sufficiently support the execution of a complex task at hand [34-39]. Therefore, health care professionals resort to workarounds to make their workflow more digestible.

Finally, the SEIPS work system component *physical environment* was incorporated into the original SEWA framework without any rationale. However, Dudding et al [25] mentioned that a busy, fast-paced environment where interruptions are constant, such as the neonatal intensive care unit, gives rise to EHR workarounds. The rationale here is “fast-paced environment” and is described as “devising workarounds to cope with the inability to, for example, update the documentation in fast-paced care environments where interruptions are constant” [25].

**Table 4:** Overview of rationales for the work system component person(s) and related included studies.

Rationales	Description	Studies
Declarative knowledge	Not knowing how to use (a part of) the EHR^a^ to accomplish a task	[20,33,34,39,43,44]
Procedural knowledge	Knowing how but not being proficient enough to use a part of the EHR to accomplish a task	[20,28,34,39,44]
Memory aid	Writing patient data down on paper (eg, keywords) or adding visual elements to parts of text in a progress note (eg, boldfacing, italicizing, or underlining) to remind oneself	[20,34,39,43,45-47]
Awareness	Storing patient data that are perceived important by the EHR user for other colleagues or patients to be noticed (frequently in a data field other than the intended field in the EHR)	[20,39,40,48]
Social norms	Formal or informal, collaborative, and cultural understandings among health care professionals leading to the creation and dissemination of workarounds (eg, mimicking workarounds devised by colleagues to accomplish a task or working around the system upon as friendly requested or enforced by a fellow clinician)	[20,29-31,45,49,50]
Trust (new)	Having insufficient trust in the (new) EHR system or its capabilities, lack of perceived usefulness, or insufficient confidence in the (completeness) of data	[20,33-39]

^a^EHR: electronic health record.

**Table 5:** Overview of rationales for the work system component EHR^a^ system and related technology and related included studies.

Rationales	Description	Studies
Usability	High behavioral user cost in accomplishing a task	[20,25,28,29,31,41,42,45,46,50-56]
Technical issues	(A part of the) EHR or ancillary technology halting, crashing, or slowing down, hampering the EHR user in accomplishing a task	[20,25,28,31-33,43,44,51-53,55-61]
Data presentation	Preferring a different data view (eg, visualization by means of charts or graphs rather than plain text)	[20,55,62]
Patient data specificity	Needing to enter or request patient data with greater or lesser specificity than offered or enforced by the EHR	[20]
Data integration (new)	EHR not providing or supporting the integration of patient data necessary for care delivery	[42,45]
Enforced actions (new)	Avoiding or overriding actions enforced by the EHR (eg, bypassing the approval process of prescribing medication or using a different user account)	[29,43,48,54,63]
Data quality (new)	Unavailability of data, disparity in data formats (eg, the same data being stored in multiple different formats in the EHR), lack of standardization, and information gaps in the EHR	[31,34-36,39,41,42,44,50,57,64-67]
Interoperability (new)	Data not able to be exchanged between health care systems or institutions (eg, causing data to be unavailable at the right moment and time)	[44,50,54,56,64,65]

^a^EHR: electronic health record.

**Table 6:** Overview of rationales for the work system component organization and related included studies.

Rationales	Description	Studies
Efficiency	Using an alternative way of accomplishing a task that improves actual efficiency	[20,29,31,34,35,37,43,46,47,55,68-70]
Data migration policy	Not having (direct) access to required historical data because of data not having been imported from previously used systems to the current EHR^a^	[20]
Enforced data entry	EHR enforcing user to enter patient data of which neither the user nor the patient has knowledge of	[20,71,72]
Required data entry option missing	EHR not offering the required data entry option (eg, 3.75 mg rather than the available options 2.5 mg or 5 mg)	[20,32,71]

^a^EHR: electronic health record.

**Table 7:** Overview of rationales for the work system component task(s) and related included studies.

Rationales	Description	Studies
Task interference	Inability to perform multiple tasks at once (eg, simultaneously treating a patient on the treatment table as well as entering patient data into the EHR^a^)	[20,61]
Commitment to patient interaction	Valuing patient interaction over computer interaction (ie, writing things down on paper and afterward entering this into the EHR)	[20,34,37,41,44,55,61,73]
Task complexity (new)	The high complexity of the tasks needing to be conducted	[34,39]

^a^EHR: electronic health record.

##### Attributes

Although several studies confirmed the previously defined attributes in SEWA, several included studies also mentioned a total of 4 new attributes (Table 8). These are concerned with whether the user is aware of using a workaround [49] (*awareness*), whether the workaround is an individual or shared practice across users [49] (*shared*), on what medium the workaround is conducted (eg, paper or computer) [34,41] (*medium*), and whether the workaround is a formal or informal practice (eg, part of a defined process or approved or promoted by management or not) [56] (*formality*).

**Table 8:** Overview of workaround attributes and related included studies.

Attributes	Description	Source
Cascadedness	Whether the workaround initiates the creation of 1 or multiple additional workarounds or is an isolated occurrence	[20]
Avoidability	Whether the workaround is required to proceed with one’s workflow or optional	[20,32,66,74]
Anticipatedness	Whether the workaround is used at known moments in time (ie, the situation in which the workaround is used is known beforehand) or used unexpectedly	[20,74]
Repetitiveness	Whether the workaround is ingrained into the workflow (ie, becomes part of daily routines) or used temporarily to overcome workflow constraints	[20,56,74]
Awareness (new)	Whether the user is aware of using the workaround	[49]
Shared (new)	Whether the workaround is a shared practice across multiple other users of the EHR^a^ or limited to 1 user	[49]
Medium (new)	On what medium the workaround is conducted (eg, paper, computer, verbal, or a combination)	[34,41]
Formality (new)	Whether the use of the workaround is approved by management and part of a defined process	[56]

^a^EHR: electronic health record.

##### Types of Impact

The previously defined types of impact in the SEWA framework were confirmed by many included studies. Multiple additional types of impact were also identified: *privacy/security*, *data quality*, *employee perception of EHR*, *financial*, *law/regulations*, and *workload* (Table 9). *Privacy/security* relates not only to the impact a workaround has on the security and privacy of the data but also to the patient and organization itself. Data quality concerns the impact on, for example, loss of data, or a lower data quality because of spelling or formatting mistakes in the data. Moreover, workarounds can have a positive or negative financial impact [58], may jeopardize laws and regulations [63,75], and have a positive or negative impact on the workload of the user [43].

**Table 9:** Overview of types of impact and related included studies.

Impact	Description	Source
Patient safety	The impact on the safety (physical and mental) of the patient	[20,28,29,41,43,46,48,53,54,58,59,67,75-77]
Effectiveness of care	The effectiveness and quality of the care process performed	[20,28,43,46,54,58,59,67]
Efficiency of care	The impact on the efficiency of the care process in terms of time and resources expended	[20,33,55,60,64,72,76]
Privacy and security (new)	Impact on the security and privacy of data related to the patient or organization	[32,39,51,52,56,63,68,75]
Data quality (new)	Impact of workarounds on data quality (eg, loss of data or decreased data quality)	[32,33,35,39,41,46,51,52,56,59,69,76]
Financial (new)	Financial implications because of the workaround	[58]
Laws and regulations (new)	Legal conflicts resulting from the use of a workaround	[63,75]
Workload (new)	An increase or decrease in workload of the EHR^a^ user resulting from the use of a workaround	[43]

^a^EHR: electronic health record.

##### Possible Scopes

Only a few studies explicitly discussed possible scopes (ie, entities impacted) of workarounds and resonated with those in the SEWA framework [41,43,53,77] (Table 10). No new possible scopes were identified.

**Table 10:** Overview of possible scopes and related included studies.

Scope	Description	Source
Patient	The workaround affects the patients in the care process	[20,43,77]
Health care professional	The workaround affects the health care professionals such as physicians, nurses, and pharmacists	[20]
Organization	The workaround affects the whole organization, including the supporting departments such as finance or legal	[20,41,53]

##### Revised Version of the SEWA Framework

**Figure 2:**
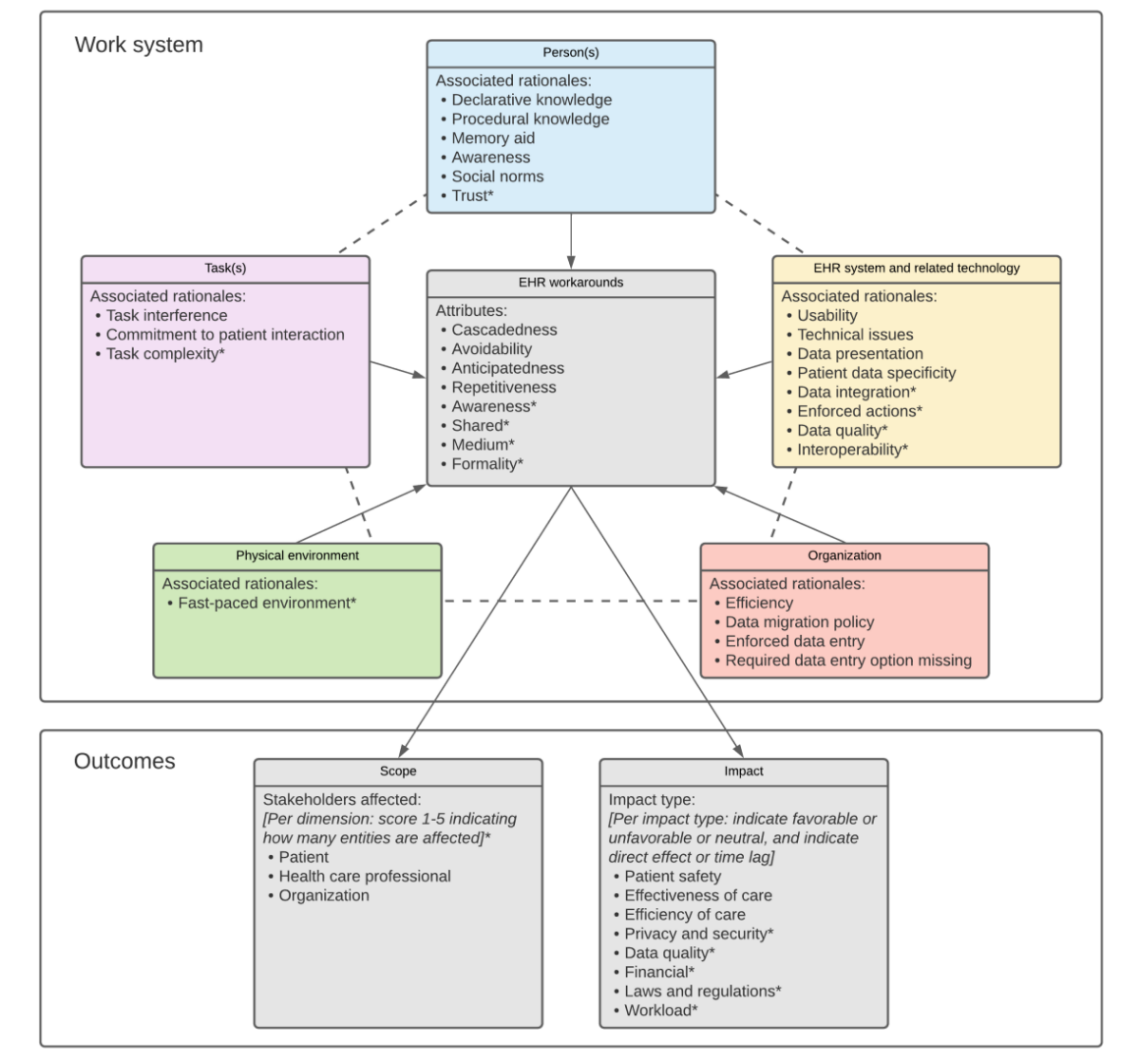
Revised SEWA framework with incorporated rationales, attributes, types of impact, and possible scopes identified in included studies. EHR: electronic health record; SEWA: Sociotechnical Electronic Health Record Workaround Analysis.

On the basis on the foregoing results, the original SEWA framework [20] was revised to incorporate new rationales, attributes, types of impact, and possible scopes identified in the included studies (Figure 2). The revised SEWA framework still comprises 2 major parts. The first part concerns the work system and its components (inspired by the SEIPS framework), [21] constituting the context in which EHR workarounds are created. The work system components now include 22 rationales (previously 15) for workaround creation, and EHR workarounds are now defined by 8 attributes (previously 4). The second part concerns the possible scope of workarounds in terms of types and number of entities affected (still 3), as well as their impact on patient safety, the effectiveness of care, the efficiency of care, and 5 newly introduced types of impact. All new items in the framework are marked with asterisks.

The recommendations [20] for using a scoring mechanism to indicate whether the impact per workaround is favorable, unfavorable, or neutral, as well as to indicate whether the impact is immediate or only observable after a certain period (*direct/time lag*) remain. However, we also recommend the inclusion of a scoring mechanism to indicate the number of patients and health care professionals and organizational units affected per applicable scope. This is in line with Carayon et al [53], who distinguished between workarounds having an impact at an individual or *team level* (eg, an entire team of nurses in a certain hospital ward). Applying a scoring mechanism allows for a more substantiated view when analyzing and prioritizing various identified workarounds for resolution.

## Discussion

### Principal Findings

A scoping review was performed to theoretically validate the SEWA framework [20] and refine and enrich it with newly identified rationales, attributes, types of impact, and possible scopes of EHR workarounds. The scoping review retrieved 737 studies, of which 62 (8.4%) were included. The included studies provided extensive support for nearly all the items included in the original SEWA framework. SEWA was revised and enriched with 7 new rationales, 4 attributes, and 5 types of impact of EHR workarounds mentioned in the included studies. The definitions of several existing rationales were also refined. As a result, SEWA is now grounded in the existing body of peer-reviewed empirical evidence on EHR workarounds published between 2010 and 2021. In addition, this revised version is likely also applicable in a wider range of health care settings as input for the original SEWA framework that came from a single comprehensive case study on EHR workarounds in an academic hospital.

### Comparison With the Literature

The results of this scoping review are in line with prior research and reviews of EHR workarounds. In an integrative review, Fraczkowski et al [78] examined nurse workarounds in EHR use. The categories defined in the review by Fraczkowski et al [78] are similar to the work system components defined in SEWA, with the exception of *usability* being a separate rationale in the SEWA framework under the work system component *EHR system and related technology* [20]. The *patient* category in the review by Fraczkowski et al [78] is defined as an impact and scope category in SEWA [20]. Finally, Fraczkowski et al [78], similar to Koppel et al [18], did not include a work system component for *person(s)* (the users of the EHR) as a category. Our scoping review is one of the few studies that investigated the entire spectrum of EHR users. On the one hand, we included studies of all types of health care professionals in primary, secondary, and tertiary care who make use of an EHR in their clinical practice, whereas other reviews merely focused on a specific population such as physicians, nurses, or secretary personnel [78]. On the other hand, we excluded studies researching workarounds in the use of barcode medication administration systems, whereas other reviews did not [78].

### Strengths and Limitations

To maximize the capture of relevant information on EHR workarounds, comprehensive and structured searches were conducted in MEDLINE, Embase, CINAHL, Cochrane, and IEEE databases. Data charting templates and analytic frames were used to extract relevant information from the reviewed studies and compare with pre-existing items in the SEWA framework.

A total of 2 research team members participated in the review process for both the title and abstract and full-text review phases, with a Cohen κ value of >0.9. This indicates an adequate interrater agreement. Despite this, our scoping review is at risk for selection bias, as we did not identify all available data, such as gray literature on EHR workarounds. There is a chance that relevant but nonincluded studies may use terminology other than the terms included in the search queries.

The broad scope of the retrieved information on EHR workarounds and the different types of studies reporting a particular issue made using a formal meta-analytic method to quantitatively assess the quality of the studies and evidence of retrieved information difficult. However, given the purpose of the scoping review to theoretically validate and refine the SEWA framework, we do not consider this limitation.

### Implications for Practice and Future Research

Multidisciplinary teams (comprising, for example, physicians, nurses, management, and EHR developers) can use the revised SEWA framework to identify, analyze, prioritize, and resolve workarounds related to EHR use more accurately. Similarly, the consequences of current and future configurations of the work system (health care professionals’ work processes and activities in relation to their EHR use) can be assessed and discussed in greater detail to determine how a design and redesign of the work system would positively or negatively affect the interaction between work system components. Finally, as workarounds are subject to gradual change (eg, personal changes in experience with the EHR, system updates to the EHR, and hospital policies), more detailed snapshots of the work system using SEWA can be taken over time and compared so as to gain valuable insights into how EHR workarounds evolve over time.

Concerning future research, EHR systems are continuously subject to technological evolution by developments in, for example, artificial intelligence, machine learning, and telemedicine. This may lead to the creation of hitherto unidentified rationales, attributes, possible scopes, and types of impact of workarounds on users, patients, and health care organizations. Similarly, more studies on EHR workarounds will continue to emerge that may report novel insights not incorporated into the revised SEWA framework. Therefore, we expect that SEWA needs a continuous process of refinement over time. This could be done by repeating the scoping review using the described search strategy, search queries, and inclusion and exclusion criteria.

In addition, although the revised SEWA framework is now theoretically validated, refined, and enriched, practical validation is still required. The same holds true when investigating its practicality. The firsthand experience from the application of SEWA in practice could yield suggestions for further improvement. A related suggestion is that although the framework helps in identifying and analyzing workarounds, a prioritization method for handling these issues is likely required, as workarounds are generally abundant in any organization, and resources to resolve them are finite. Therefore, the framework could benefit from being extended with prioritization mechanisms and weighting factors for deciding which workarounds require priority. Similarly, the framework could be translated into a practical tool such as a scoring matrix to facilitate use by practitioners.

Finally, the applicability of the SEWA framework could be explored for systems other than EHRs (eg, enterprise resource planning, customer relationship management, and content management) and in other settings (eg, nonacademic hospitals and general practitioner practices) and even in other industries (eg, financial services and manufacturing) after appropriate validation. Although SEWA has an explicit focus on EHRs used in health care, we expect many of the described workaround rationales and attributes to be applicable to other systems, settings, and industries.

## Appendix

Multimedia Appendix 1 Descriptive data template that was captured per included study.

Multimedia Appendix 2 Analytic frame with workaround-related data captured per study.

## Abbreviations

EHR: electronic health record
PRISMA: Preferred Reporting Item for Systematic Reviews and Meta-Analyses
SEIPS: Systems Engineering Initiative for Patient Safety
SEWA: Sociotechnical Electronic Health Record Workaround Analysis
