# Supplementary material for: Workarounds in Electronic Health Record Systems and the Revised Sociotechnical Electronic Health Record Workaround Analysis Framework: Scoping Review
Source: J Med Internet Res. 2022 Mar 15;24(3):e33046. doi: 10.2196/33046 (PMC8965666; doi:10.2196/33046)
Supplement: Multimedia Appendix 1 [file jmir_v24i3e33046_app1.docx]

## Appendix A

Appendix A: Descriptive data template that was captured per included study.

| **Author and date** | E.g., Name, Name & Name (2019) |
| --- | --- |
| Title of study | E.g., Example Title |
| Year of publication | E.g., 2019 |
| Study setting | E.g., Primary care |
| Experience with EHR | E.g., 2 years |
| Functionalities of EHR studied | E.g., … |
| Type of population and size | E.g., … |
| Methods | E.g., Time-and-motion study |
