# Supplementary material for: Workarounds in Electronic Health Record Systems and the Revised Sociotechnical Electronic Health Record Workaround Analysis Framework: Scoping Review
Source: J Med Internet Res. 2022 Mar 15;24(3):e33046. doi: 10.2196/33046 (PMC8965666; doi:10.2196/33046)
Supplement: Multimedia Appendix 2 [file jmir_v24i3e33046_app2.docx]

## Appendix B

Appendix B: Analytic frame with workaround-related data captured per study.

| **Author and date** | E.g., Name, Name & Name (2019) |
| --- | --- |
| Categories of workarounds |  |
| *Work components* | … |
| *Rationales* | … |
| *Attributes* | … |
| *Consequences* | … |
| *Scope* | … |
| Other components | … |
| Limitations of study | … |
| Other details of study | … |
